# Editorial: In celebration of women in science: cellular biochemistry

**DOI:** 10.3389/fmolb.2025.1595189

**Published:** 2025-03-26

**Authors:** Graca Soveral, Catalina Alba Soto, Cecilia Giulivi

**Affiliations:** ^1^ Research Institute of Medicines (iMed.Ulisboa), Faculty of Pharmacy, Universidade de Lisboa, Lisbon, Portugal; ^2^ Institute of Research in Medical Microbiology and Parasitology (IMPaM; UBA-CONICET), School of Medicine, University of Buenos Aires, Buenos Aires, Argentina; ^3^ School of Veterinary Medicine, University of California Davis, Davis, CA, United States

**Keywords:** women, stem, science, discovery, gender-gap

The field of molecular biosciences has long been driven by curiosity, innovation, and an unyielding quest for knowledge. Yet, despite their groundbreaking contributions, women remain underrepresented in scientific research, constituting less than 30% of researchers worldwide (UNESCO, 2020). This stark reality highlights the persistent challenges posed by gender biases and stereotypes, which continue to deter many women from pursuing careers in STEM (Schneegans et al., 2021; Economic and Forum, 2023). However, progress is being made, and interventions aiming at closing the gender gap, as mentoring programs, scholarships, and initiatives such as the *Women in Science* series by *Frontiers in Molecular Biosciences* play a crucial role in amplifying the voices of women researchers and showcasing their invaluable contributions to science.

The latest Research Topic highlights diverse and cutting-edge research in cellular biochemistry, spanning Research Topic from disease mechanisms to novel therapeutic approaches. Dr. Alejandra Tomas and her team explore the variability in patient responses to incretin therapy for Type 2 diabetes, shedding light on the molecular and cellular factors influencing treatment success (Austin and Tomas). Meanwhile, Rosa Catapano and colleagues investigate the role of the ZNF224 protein in chronic lymphocytic leukemia, uncovering its potential as a prognostic marker and its link to the NF-kB survival pathway (Catapano et al.).

Beyond human disease, Fechtali-Moute et al. delve into the life cycle of *Acanthamoeba castellanii*, a free-living amoeba that can cause severe infections. Their study identifies enzyme treatments that promote excystment and rapid trophozoite proliferation, paving the way for potential therapeutic interventions (Fechtali-Moute et al.). Estelle Sontag’s research team examines the role of PP2A methylation in tight junction assembly, providing new insights into cell polarity and epithelial barrier function (Schuhmacher et al.). Finally, Catarina Pimpão’s review highlights the emerging role of aquaglyceroporin modulators in treating diseases related to energy homeostasis, demonstrating the translational impact of cellular biochemistry (Pimpão et al.).

These studies advance scientific understanding and underscore women’s critical role in driving innovation and discovery. By celebrating their contributions, we reaffirm the importance of gender equality in science and encourage future generations of women to break barriers and pursue careers in STEM. Science thrives on diversity, and by fostering inclusivity, we ensure a more robust and dynamic research community—one that benefits all of humanity.
